# Theranostic imaging of liver cancer using targeted optical/MRI dual-modal probes

**DOI:** 10.18632/oncotarget.15642

**Published:** 2017-02-23

**Authors:** Qingshan Chen, Wenting Shang, Chaoting Zeng, Kun Wang, Xiaoyuan Liang, Chongwei Chi, Xiao Liang, Jian Yang, Chihua Fang, Jie Tian

**Affiliations:** ^1^ Department of Hepatobiliary Surgery, Zhujiang Hospital, Southern Medical University, Guangzhou 510280, China; ^2^ Key Laboratory of Molecular Imaging, Institute of Automation, Chinese Academy of Sciences, Beijing 100190, China; ^3^ Beijing Key Laboratory of Molecular Imaging, Beijing 100190, China

**Keywords:** liver cancer, MRI/optical, preoperative diagnosis, intraoperative navigation, dual-modality

## Abstract

The accurate preoperative detection and intraoperative navigation afforded by imaging techniques have had significant impact on the success of liver cancer surgeries. However, it is difficult to achieve satisfactory performance in both diagnosis and surgical treatment processes using any single modality imaging method. Here, we report the synthesis and characteristics of a novel dual-modality magnetic resonance imaging (MRI) and near-infrared fluorescence (NIRF) probe and verify its feasibility in nude mouse models with liver cancer. The probes are comprised of superparamagnetic iron oxide (SPIO) nanoparticles coated with liposomes to which a tumor-targeted agent, Arg-Gly-Asp peptides (RGD), and a NIRF dye (indocyanine green, ICG) have been conjugated. Specific targeting, biodistribution, and the imaging ability of the probes for MRI-NIRF were examined. Furthermore, we applied the dual-modality methodology toward the preoperative diagnosis and intraoperative guidance of radical resection in mouse models with both orthotopic liver tumors and intrahepatic tumor metastasis. The study demonstrated that both MRI and fluorescent images showed clear tumor delineation after probe injection (SPIO@Liposome-ICG-RGD). The contrast-to-noise ratio obtained from MRI was 31.9 ± 25.4 at post-injection for the preoperative diagnosis, which is helpful for detecting small tumors (0.9 ± 0.5 mm). The maximum tumor to background ratio of NIRF imaging was 2.5 ± 0.3 at 72 h post-injection for effectively capturing miniscule tumor lesions (0.6 ± 0.3 mm) intraoperatively. The novel MRI-NIRF dual modality probes are promising for the achievement of more accurate liver tumor detection and resection.

## INTRODUCTION

Liver cancer is one of the most common malignant cancers worldwide with an estimated incidence of 782,500 cases in 2012, of which roughly half arose in China [1]. Approximately 40 % of patients with liver cancer are diagnosed in the early stage and have a 5-year survival rate of about 28 %. Survival decreases to 10 % and 3 % for patients who are in the regional and distant stages of the disease, respectively [2]. Therefore, there is an urgent need for accurate preoperative diagnosis and assessment, along with optimization of the treatment scheme.

Surgical resection is one of the major therapeutic methods for liver cancer; however, its 5-year risk of recurrence exceeds 70 % [3], with micro-vascular invasion and micro-residual lesions representing the main risk factors for postoperative recurrence [4]. Therefore, it is vital for surgeons to realize radical resection to facilitate patient recovery.

Diverse imaging modalities for liver cancer currently exist, such as positron emission tomography-computed tomography (PET-CT), magnetic resonance imaging (MRI), and fluorescence molecular imaging (FMI), each of which carries specific advantages and disadvantages. However, no single imaging modality has to date been able to simultaneously provide the entirety of preoperative structural information and intraoperative local functional information for liver cancer required in clinical practice.

MRI is commonly applied in clinical liver cancer diagnosis owing to its excellent soft tissue contrast and spatial resolution [5]. Intraoperative near-infrared fluorescence (NIRF) imaging is also frequently applied for the guidance of various tumor resections because of its unique sensitivity and real-time ability [6]. Therefore, the development of an MRI-NIRF dual modality molecular imaging probe might have the potential to combine the advantages of both imaging technologies and enable better theranostic performance for patients with liver cancer.

In our research, we synthesized and tested a novel MRI-NIRF molecular imaging nanoprobe, SPIO@Liposome-ICG-RGD. The arginine-glycine-aspartic acid (RGD) peptide can bind specifically to the integrin αvβ3 receptor, which is the most important integrin for angiogenesis [7], which in turn plays a crucial role in the pathological development of many solid tumors including liver cancer [8]. The superparamagnetic iron oxide nanoparticles (SPIO NP) have the characteristics of lower toxicity, high sensitivity, and excellent biocompatibility, and were the first T2 contrast agent for MRI to be used clinically [9]. SPIO, as for the commercial product Feridex®, was approved by the U.S. Food and Drug Administration (FDA) for the diagnosis of liver lesions [10] and are selectively taken up by Kupffer cells in the liver, wherein rapid dephasing of neighboring proton spins leads to a shortening of the spin-spin (T2) relaxation times. In contrast, liver tumors that lack Kupffer cells cannot take up the SPIOs effectively. Therefore, T2-weighted images of normal tissues show darkening [11]. Indocyanine green (ICG) is the only near infrared reflection (NIR) organic dye approved by the FDA for human medical imaging and diagnosis in clinical practice [12].

Here, we found that the MRI and fluorescence images showed clear tumor delineation at post-injection and 72 h post-injection, respectively. Therefore, the targeted dual-modality imaging probe was conducive to improving preoperative surgical planning and intraoperative fluorescence-guided surgery.

## RESULTS

### Characterization of SPIO@Liposome-ICG-RGD

**Figure 1:**
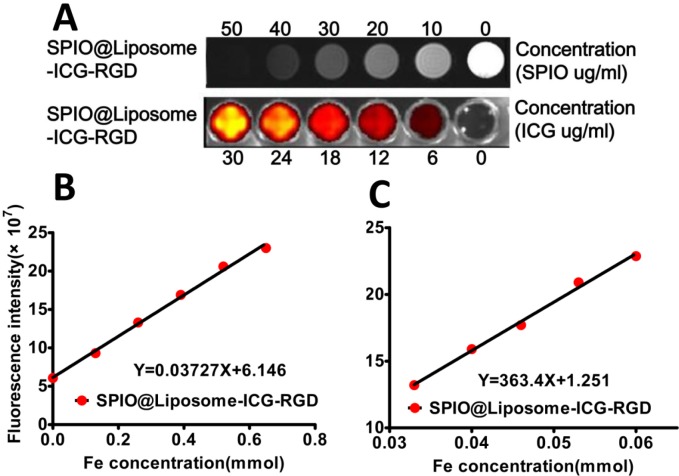
MRI and NIRF signal evaluation for SPIO@Liposome-ICG-RGD **A**. The concentration of the probe was successively diluted from the left to right wells. For MRI, the negative contrast effect is clearly demonstrated with the decrease of SPIO concentration. However, for NIRF imaging, the optical signal decreases with diminishing ICG concentration. **B**. A linear correlation is observed between the fluorescence intensity and the probe concentration, whereas **C**. an inverse linear correlation can be seen between the T2-weighted MRI signal and the probe concentration. The R2 of the probe is 363.4 mM^−1^ s^−1^.

The morphology and size of the SPIO@Liposome-ICG-RGD probes were characterized using transmission electron microscopy (TEM) and dynamic light scattering, respectively. The TEM image revealed that the typical spherical morphology of the probe exhibited an approximate average diameter of 126 ± 21 nm (Supplementary Figure 1). Thus, the size (< 150 nm) of the probe is particularly favorable for tumor-targeting *in vivo* [13]. Spectroscopic measurement of the probe showed that the NIR signal peaked at an emission wavelength around 810 nm. With constant SPIO (250 mg/mL), different supplied amounts of ICG (25–150 μg/mL) yielded a similar absorbance between 780 nm and 830 nm in the UV-vis absorption spectra, which revealed that the probe was suitable for NIRF imaging (Supplementary Figure 2). With the increase in the probe concentration, the T2-weighted MRI signal increased gradually (negative contrast effect) and the optical signal also increased (Figure 1A). Both signals types showed a linear correlation (MRI: r2 = 0.9954; NIRF: r2 = 0.9962) with the probe concentration (Figure 1B and Figure 1C). This suggested that the fluorescence was protected from the bleaching of SPIO because of the interval produced by the liposome. The relaxivity of the probe was 363.4 mM^−1^ s^−1^, which was approximately 3 times better than that of the FDA-approved drug Feridex® for the detection of liver lesions [14]. No significant decrease of cell viability was observed when cells were incubated with any of the three NPs at a concentration range from 0–100 μg/mL (Fe_3_O_4_ concentration) (Supplementary Figure 3), which demonstrated their good biocompatibility. Furthermore, the optical stability assessment indicated that the probe was stable in mosue serum over 96 h (Supplementary Figure 4 and Supplementary Figure 5). In the integrin binding assays, the targeted probe showed a 4.1 ± 1.1-fold (P < 0.05) increase in the uptake of SPIO when incubated with HepG2 cells (Supplementary Figure 6A) and a 2.6 ± 0.5-fold increase in fluorescence intensity (P < 0.05) compared with the non-targeted control probe (Supplementary Figure 6B). This double verification confirmed the specific targeting ability of the targeted probes. Together, these results indicated that the optical/MRI probe was able to function efficiently in dual modality imaging.

### Evaluation of MRI contrast enhancement *in vivo*

**Figure 2:**
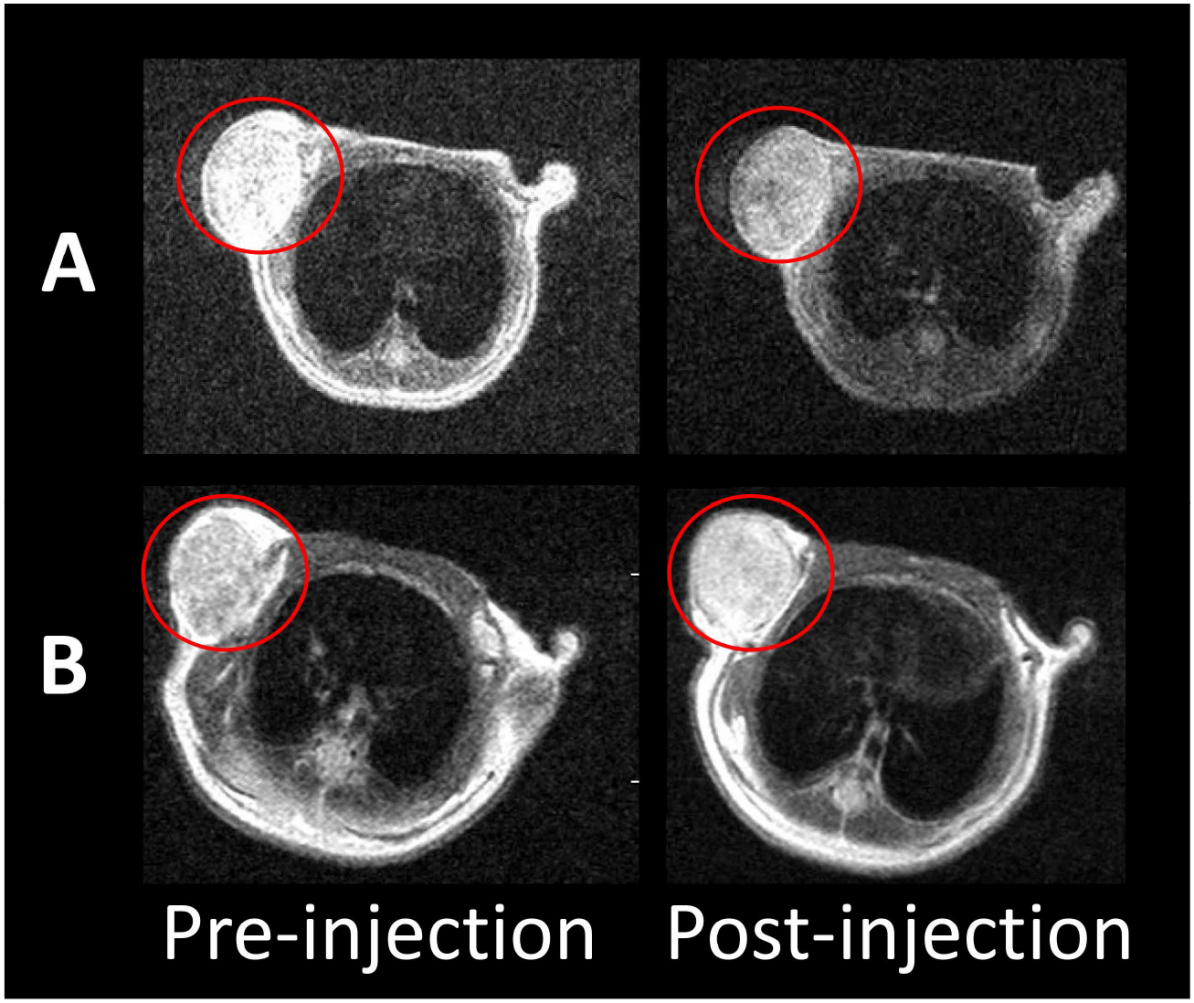
*In vivo* T2-weighted MR imaging before and after the injection of SPIO@Liposome-ICG-RGD **A**. or SPIO@Liposome-ICG **B**.

**Figure 3:**
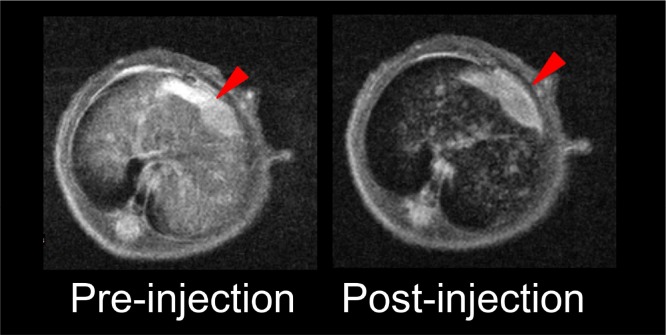
*In vivo* continuous observations of the orthotopic liver cancer model administrated with SPIO@Liposome-ICG-RGD The contrast-to-noise ratio (CNR) of SPIO@Liposome-ICG-RGD was calculated as CNR = (SI_liver_−SI_tumor_)/SI_noise_.

The quantification of MRI contrast SI between the liver and the tumor was acquired by calculating the contrast-to-noise ratio (CNR) as CNR = (SI_liver_−SI_tumor_)/SI_noise_ [14]. *In vivo* MR imaging was performed prior to and after injection, respectively. For mice receiving the targeted probe, the spatial distribution of the decreased pixels was heterogeneous and occurred mainly in the periphery of the tumor (Figure 2A). For mice receiving the non-targeted probe, the pixel signal decrease was much less pronounced and scattered within the tumor rim (Figure 2B). The decreased signal in the tumors was 24.7 ± 1.5 % after the administration of the targeted probe, whereas only a 5.3 ± 2.0 % (P < 0.05) decrease was observed in the non-targeted group. In the orthotopic liver tumor nude mouse model (n = 5) (Figure 3), we found marked signal reductions in the normal liver tissue following the injection. The maximum CNR was about 31.9 ± 25.4 at post-injection. To our knowledge, the CNRs were found to be only 146.6 ± 18.9 for Feridex® [10]. These data indicated that MRI scanning should be performed after the injection of the probes for preoperative assessment.

### *In vivo* optical imaging and histology examination

**Figure 4:**
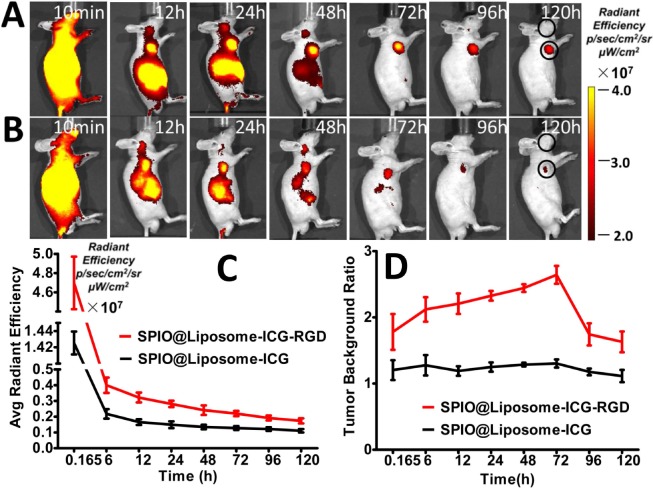
*In vivo* comparison of the biodistribution of SPIO@Liposome-ICG-RGD and SPIO@Liposome-ICG **A, B**. *In vivo* continuous observations (120 h) of liver cancer xenografts administrated with SPIO@Liposome-ICG-RGD (A) or SPIO@Liposome-ICG (B) using FMI. Black circles indicate the regions of interest for calculating the tumor to background ratio (TBR) in each time point. The quantification of fluorescence intensity at the tumor sites reveals a higher accumulation of SPIO@Liposome-ICG-RGD at all observation points **C**. Comparison of TBR profiles of the two probes. The peak and maximum difference both occurred at 72 h post-injection, suggesting the optimal surgical window time **D**. Experiments were run in triplicate.

After continuous observation over 120 h, fluorescence imaging indicated a significant accumulation of both probes in tumor lesions up to 120 h. However, the SPIO@Liposome-ICG-RGD probe demonstrated better optical contrast than the non-targeted probe in the tumor region starting at 24 h post-injection (Figure 4A-4B). After the initial distribution period (< 12 h), the majority of the optical signals were observed in the tumor and abdominal areas and gradually faded away 24 h post-injection. These results suggested that the probes were eliminated through the liver and spleen, which aligned with our expected 100–150 nm probe size [13]. Furthermore, the quantification of radiant efficiency showed that SPIO@Liposome-ICG-RGD displayed a stronger optical signal in tumor lesions at all observation points (Figure 4C), verifying its excellent *in vivo* targeting ability for integrin αvβ3-overexpressing tumors. Notably, the tumor-to-background ratio (TBR) peak of SPIO@Liposome-ICG-RGD (2.6 ± 0.1) appeared at 72 h post-injection and was about 2-fold higher than that of SPIO@Liposome-ICG (1.3 ± 0.1) at the corresponding time (Figure 4D). The *ex vivo* comparisons of the biodistribution between the targeted and untargeted probes were performed on the major organs (tumor, heart, liver, spleen, lungs, and kidneys) at 72 h post injection, which also demonstrated the higher tumor uptake of the SPIO@Liposome-ICG-RGD group (Supplementary Figure 7). These data confirmed SPIO@Liposome-ICG-RGD tumor specificity and indicated that intraoperative navigation should be performed 72 h following probe injection.

### Dual-modality imaging using the SPIO@Liposome-ICG-RGD probe detects orthotopic liver tumors

**Figure 5:**
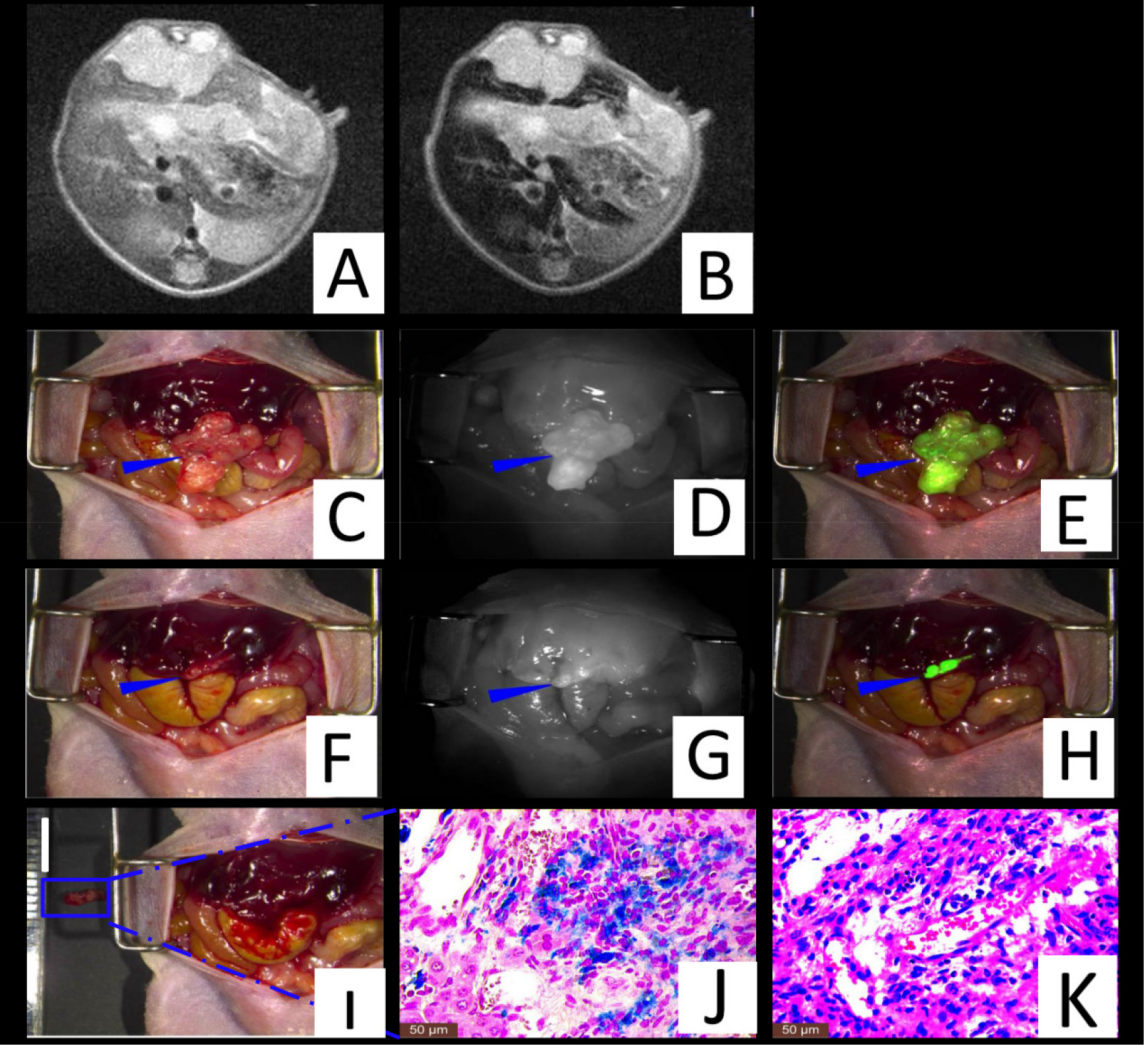
Theranostic imaging in the orthotopic liver tumors models The MRI image before SPIO@Liposome-ICG-RGDs injection **A**. The MRI signal is obviously decreased in normal liver tissue (CNR: 34.2 ± 9.1) after targeted probe injection **B**. Surgical guidance by intraoperative FMI-NIR (fluorescence molecular imaging system) **C**. The implanted liver tumor tissue (blue arrow) exhibits obvious contrast (TBR: 2.4 ± 0.3) in color and texture with normal liver tissues **D**. The merge image of color and fluorescence demonstrates the excellent contrast **E**. The residual tumor node (blue arrow) after the first operation **F**. The residual tumor node exhibits obvious contrast (TBR: 2.5 ± 0.3) in color and texture with normal liver tissues **G**. The merged color and fluorescence image demonstrates the excellent contrast in the residual tumor node **H**. Identification of the residual tumor (0.6 ± 0.3 mm) after the initial resection **I**. Prussian blue staining confirmation of the targeting ability of SPIO@Liposome-ICG-RGDs **J**. HE staining confirmation of the liver tumor tissue **K**. Experiments were run in triplicate.

Orthotopic liver tumor nude model mice (n = 5) were established by two experienced surgeons. We initially performed MRI scanning to determine the characteristics of the primary liver tumors including the location, size, and number. A marked MRI signal decrease was observed in normal liver tissue subsequent to probe delivery (Figure 5A-5B). The maximum CNRs were found to be 34.2 ± 9.1 after the injection of SPIO@Liposome-ICG-RGD. Via intraoperative fluorescence navigation through laparotomy in another model, we observed that the fluorescent signal of the tumor was much higher than that of the normal liver tissue in the orthotopic models. Under the fluorescence surgical navigation system, we easily resected the large tumor tissue mass, which exhibited a TBR at 2.4 ± 0.3 fold (Figure 5C-5F). After the first operation, the miniscule residual tumor tissue (0.6 ± 0.3 mm) in the surgical area was still much brighter than the normal liver tissue, which raised the concern that the operation was not sufficient to effect radical resection. We further removed the miniscule residual tumor under the optical image with the TBR at 2.5 ± 0.3 fold (Figure 5G-5I). Finally, the above surgical samples were confirmed by both Prussian blue staining and HE staining (Figure 5J-5K).

### Dual-modality imaging using the SPIO@Liposome-ICG-RGD probe detects primary liver tumors with intrahepatic metastasis

**Figure 6:**
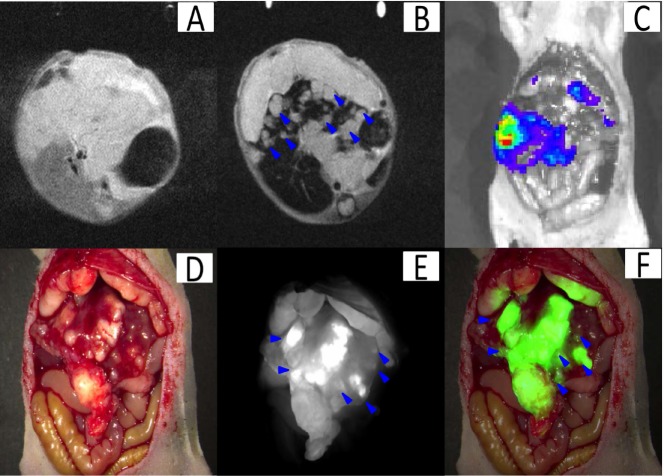
Theranostic imaging in the orthotopic liver tumors with intrahepatic metastasis The MRI image before SPIO@Liposome-ICG-RGDs injection **A**. The MRI signal is obviously decreased in normal liver tissue (CNR: 14.6 ± 9.9) after targeting probe injection and the disseminated tumor nodes (0.9 ± 0.5mm) (blue arrow) can be clearly defined **B**. The presence of liver tumors as confirmed by bioluminescence imaging **C**. Surgical guidance by intraoperative FMI-NIR **D**. The implanted liver tumor tissue (0.7 ± 0.3 mm) (blue arrow) exhibits obvious contrast (TBR: 2.3 ± 0.5) in color and texture with normal liver tissues **E**. The merged color and fluorescence image demonstrates the excellent contrast **F**. Experiments were run in triplicate.

A total of 5 liver tumor nude mouse models with intrahepatic metastasis were established based on the orthotopic liver tumor model for extending its growing time. The preoperative MRI scanning assisted with not only detecting the large- but also the smaller tumors (0.9 ± 0.5 mm) with a CNR of 14.6 ± 9.9 (Figure 6A-6B). The bioluminescence imaging assisted with tumor confirmation (Figure 6C). After the preoperative assessment, we aggressively detected the tiny tumors under the fluorescence surgical navigation system. The small tumors (0.7 ± 0.3 mm) were much brighter than the surrounding normal liver tissue (Figure 6D-6F) with the TBR at 2.3 ± 0.5. These results might have profound value in clinical practice. For example, for patients with liver cancer exhibiting similar degrees of metastasis, subsequent to MRI scanning surgeons might recommend that the patient not proceed with surgery, which might reduce their physiological and psychological stress and therefore might represent their best overall choice.

## DISCUSSION

Imaging has played a vital role in the diagnosis of liver cancer. Multi-modal imaging, which can integrate the advantages of single image models, is popular with researchers [15]. However, there are few successful reports on simultaneously achieving “assessment preoperatively and guided intraoperatively” for liver cancer in clinical management [16]. This strategy would allow the surgeons to understand the number, location, and size of the liver tumors preoperatively and to precisely remove them using imaging-guidance intraoperatively, which would likely greatly improve the accuracy of resection, avoid futile surgical procedures, and promote the enhanced recovery of patients with liver cancer.

For example, a CT/fluorescence probe for studying atherosclerotic plaques was reported by Ding et al. [17], and Sampath et al. synthesized a PET/near-infrared agent for staging breast cancer and guiding the subsequent intraoperative resection [18]. However, compared to the ionization radiation of CT and the high costs of PET-CT, MRI is an ideal model for acquiring overall information and optical imaging is a perfect complement for MRI to obtain real-time intraoperative data owing to its excellent sensitivity [19].

There are, however, few probes available for liver cancer. It is known that the liver is the main organ for probe elimination, which induces a nonspecific background signal of many agents and hinders the development of a probe for liver lesions. Jin et al. reported a bifunctional probe for targeting and imaging liver cancer, but this was only achieved *in vitro* [20]. In addition, Hui et al. only achieved dual-modality imaging in heterotopic liver tumors [21].

SPIO can be absorbed to a large degree by Kupffer cells in normal liver tissues; however, liver tumors lacking these cells contain few probes despite attaining the targeting effect after injection. These probes at tumor sites were unable to provide adequate MRI signals; however, along with the metabolism of the probes by the liver; the provided signal was sufficient for fluorescence detection owing to the extreme sensitivity of optical imaging. Thus, in our research, we combined the spatial resolution of MRI and the excellent sensitivity of optical imaging, utilized the advantages of the characteristics of liver metabolism, and undertook MRI imaging and optical imaging at different time points. To our knowledge, this is the first study to successfully describe the advantages of the characteristics of probe metabolism by the liver and achieve the “assessment preoperatively and guided intraoperatively” in liver cancer models.

In this study, we conducted MRI and fluorescence experiments both *in vitro* and *in vivo*. For the specific targeting test, we verified a marked increase in the cellular uptake of ICG as measured by the fluorescence intensity in the targeted probe group, as well as a marked increase of SPIO as determined ICP-MS. These experiments verified the efficacy of our probes in specially targeting the αvβ3 receptor of HepG2 cells. These observations can be explained as follows: upon specific binding of the RGD-peptide to integrin αvβ3, the RGD-containing nanomaterials would be internalized into the cells by receptor-mediated endocytosis. The RGD peptide then would combine with the peptide ligand for neuropilin-1, giving rise to selective tumor vascular targeting and facilitating the penetration of imaging agents into the tumor [22, 23]. The linear relationship between the probe concentration and the optical intensity indicated that ICG was successfully loaded into the lipid layer of the SPIO@Liposome. The R2 relaxivity for the SPIO@Liposome-ICG-RGD was 363.3 mM^−1^ s^−1^, which was superior to the 243.3 mM^−1^ s^−1^ of the commercial MRI agent Feridex® [10]. We propose that the T2 relaxivity of the PEGylated SPIO@Liposome nanoparticles was amplified by the contribution of water exchange on the transverse relaxation time owing to the effect of PEG attached to the bilayer and in the SPIO coating [24]. For the *in vivo* MRI experiment, the maximum CNR was 31.9 ± 25 at post-injection. The occurrence of the maximum CNR at this time point was mainly the consequence of the sensitivity of MRI scanning and the tendency of our probe to be readily eliminated by the liver owing to its size. We determined that the optimal time point for the maximum TBR was 72 h post-injection for optical imaging. The TBR was 2.6 ± 0.1 in the SPIO@Liposome-ICG-RGD group, which was about 2-fold compared to the TBR value of 1.3 ± 0.1 in the control group, suggesting that our probes have a high efficiency of tumor targeting ability and were sufficient for intraoperative surgical guidance procedures. In the orthotopic model, the probe was able to assist in obtaining the information of location and size of the tumor preoperatively and to highlight the otherwise readily omitted tiny tumor (0.6 ± 0.3 mm) intraoperatively. In the intrahepatic metastasis model, the preoperative assessment was able to aid in staging the liver cancer according to the preoperative MRI scanning image and in the proposal of a reasonable treatment scheme, which would likely have considerable impact on clinical practice.

The ultimate aim of our studies was to improve the practical application of this system. The SPIO@Liposome-ICG-RGD exhibits an attractive perspective toward clinical application for the following reasons. First, SPIOs represent the first nanoparticle MRI contrast agents used clinically [9], and have excellent biocompatibility and R2 relaxivity. Second, ICG is the only NIR dye approved by the FDA for diagnosis in clinical applications [12]. Furthermore, RGD is a bio-polypeptide, which facilitates its incorporation into the liposomes that are primarily composed of lecithin and cholesterol. Together, these traits suggest a potential wide breadth of clinical application. In addition, both ICG and SPIO are excellent photothermal agents and can be used in the photothermal treatment process [25, 26].

## MATERIALS AND METHODS

### Cell lines

The high αvβ3 receptor-expressing human liver cancer (HepG2) was purchased from the Academy of Military Medical Sciences (China) and cultured in Dulbecco's modified Eagle's medium (Hyclone, Beijing, China) supplemented with 1 % penicillin/streptomycin (Gibco, China) and 10 % fetal bovine serum (Gibco). The cultured cells were maintained at 37°C and 5 % CO_2_ in a tissue culture incubator.

### Animal experiments

The 6 to 8-week-old Balb/c male nude mice which had HepG2 were obtained from the Laboratory Animal Center of the Chinese Academy of Medical Sciences. All animal experiments were conducted in compliance with the regulations established by the Institutional Animal Care and Use Committee of Southern Medical University. All animal procedures were implemented with isoflurane gas anesthesia (3% isoflurane-air mixture). The subcutaneous liver tumor mice models were established by subcutaneously injecting 5 × 10^6^ HepG2 cells. The orthotopic liver tumor mice models were established by injecting 5 × 10^6^ cells into the liver via laparotomy. The animals were administered the probes at 10 mg/kg (total amount of SPIOs) (n = 5) for the *in vivo* MRI and optical studies.

### Synthesis of SPIO@Liposome-ICG-RGD

**Figure 7:**
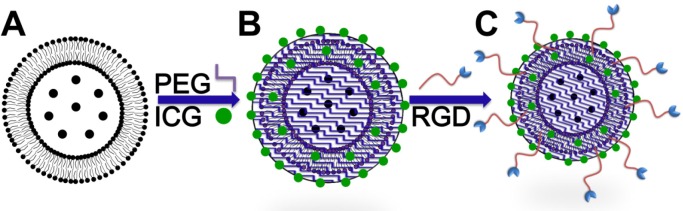
Schematic diagram for the synthesis of SPIO@Liposome-ICG-RGD **A**. SPIO nanoparticles was coated with liposome (SPIO@Liposome). **B**. ICG molecules were loaded into the lipid layer of magnetic liposomes (SPIO@Liposome-ICG). **C**. RGDs were conjugated to obtain the SPIO@Liposome-ICG-RGD probes.

Figure 7 illustrates the procedures used for preparing the SPIO@Liposome-ICG-RGD probes. The SPIO cores (Fe_3_O_4_ NPs) were synthesized as described previously [27]. The hydrodynamic diameter of the Fe_3_O_4_ NPs was 26 ± 4 nm based on dynamic light scattering with a zeta potential of −20.2 mv (Malvern Instruments Zetasizer Nano ZS, Malvern, UK). The SPIO@Liposome was produced by the film method followed by extrusion as described by Sabate et al. [28] with some modification. Briefly, as an increased content of cholesterol could reduce the uptake of liposomes by the liver [29], we adjusted the soybean lipid phosphatidylcholine and cholesterol, at a molar ratio of 1:1, to the amounts required to meet a lipid concentration of 10 mM, which was dissolved in a mixture of chloroform and ethanol(volume ratio:3:1). The solvent was evaporated with a rotary evaporator (Rotavapor r-144, BUCHI, New Castle, DE, USA) under reduced pressure at 40°C and the lipid film was produced in a round bottom flask. After the removal of chloroform, the lipid film was mixed with 250 μg/mL SPIO water suspension to generate a 5 mL solution. A total of 30 min of bath sonication was performed prior to extrusion for completing the hydration of the lipid film. The extrusion (ATS Extruder, Cambridge, ON, Canada) was performed 4 times using polycarbonate membranes (Whatman, Maidstone, UK) with a pore diameter of 100 nm. Then, the SPIO@Liposome nanoparticles were coated with polyethylene glycol (PEG) MW-5000 using a dual solvent exchange method. An ICG water solution (25, 50, 75, and 150 mL) at a concentration of 1 mg/mL was then mixed with 1 mg/mL SPIO@Liposome for 1 mL probe sample to make 1 mL. The mixture was then placed on a shaker and incubated overnight at room temperature. The unloaded ICG molecules and SPIOs were removed by ultracentrifugation at 10,000 rpm. The RGD (Arg-Gly-Asp, 5 mg, 98%, USA) peptide was combined with the SPIO@Liposome-ICG nanoparticles using the NHS-EDS method and vibrated for 6 h. The targeted probe was stored at room temperature in silver paper to protect it from light. For *in vivo* imaging, 250 μg SPIO@Liposome-ICG-RGD (total amount of SPIO) was used for each mouse, which contained 12.5 μg ICG dye molecules.

### Cell cytotoxicity assays

The HepG2 cells were used for the cytotoxicity assays. The cellular cytotoxicity of the SPIO, SPIO@Liposome-ICG, and SPIO@Liposome-ICG-RGD probes were evaluated by the 3-(4, 5-dimethylthiazol-2-yl)-2, 5-diphenyltetrazolium bromide (MTT) assay (Promega, Shanghai, China). The HepG2 cells were cultured in 96-well microtiter plates at 10,000 cells per well. After 24 h, the cells were washed with phosphate buffered saline (PBS) and the medium was replaced by fresh medium with various concentrations of SPIO, SPIO@Liposome-ICG, or SPIO@Liposome-ICG-RGD (10, 20, 40, 60, 80, and 100 μg/mL, Fe). An equivalent volume of fresh medium (200 μL/well) was added to the control wells. After 24 h incubation, the treated cells were added to PBS. About 100 μL of fresh medium and 10 μL MTT solution (5 mg/mL in PBS) were added to each well and then cultured for another 4 h. After the removal of the old medium, 120 μL dimethyl sulfoxide was added to each well and the absorbance at 600 nm was read using a microplate reader to calculate relative cell viabilities.

### Integrin binding assays

To verify the targeted specificity of SPIO@Liposome-ICG-RGD for HepG2 liver cancer cells, *in vitro* cell uptake experiments were conducted. Approximately 1 × 10^5^ HepG2 cells were cultured in 10 mL of culture medium for 24 h. Then, the SPIO@Liposome-ICG or SPIO@Liposome-ICG-RGD (iron concentration, 0.03 mM) was added for another 4 h. After washing three times with cell culture medium and twice with PBS, another 5 mL cell culture medium was added. The NIR fluorescence images (ProEM 1024B Excelon, Princeton Instruments, Trenton, NJ, USA) were acquired by the electron-multiplying CCD (EMCCD) and the noise to background ratio was analyzed [30]. In addition, the iron content per cell was analyzed using inductively coupled plasma mass spectrometry (ICP-MS) in the same sample. The number of cells was obtained using a Neubauer counting chamber.

### *In vivo* tumor fluorescence imaging

*In vivo* fluorescence imaging was performed on ten mice with subcutaneous liver tumors >5 mm in diameter. About 250 μL SPIO@Liposome-ICG or SPIO@Liposome-ICG-RGD (n = 5/group) was delivered by the tail vein. The IVIS Spectrum Imaging System (PerkinElmer, Rodgau, Germany) was used to monitor the change in the fluorescence signal of the mice at 10 min and at 12, 24, 48, 72, 96, and 120 h post-injection with excitation and emission wavelengths of 780 nm and 830 nm, respectively. Imaging data were analyzed with IVIS Living Image 3.0 software. The region of interest (ROI) was selected for measuring the mean fluorescence intensity (MFI) of the tumors. The TBR was acquired by the MFI of the tumor area divided by the corresponding body background area.

### *In vivo* tumor MR imaging

The *in vivo* MRI tests were performed on a 1.0-T MRI system (Aspect, Shoham, Israel). The T2-weighted fast spin echo imaging sequence with a TR of 2394 ms, TE of 22.2 ms, field of view of 30 mm × 30 mm, and slice thickness of 1 mm was used to obtain MR images. The MRI contrast change in the tumor following injection of the targeted probe was quantitatively calculated using the ROI method with Mxliteview (Philips). Averaged MRI signal intensities of the ROI were calculated from the tumor and corresponding noise areas. The normalized signal intensities (SI) were obtained by comparing SIpre and SIpost of the tumor ROI. The mean MRI contrast change was calculated from five MRI image slices of the entire tumor.

### Probe biodistribution and histologic examination

The mice were sacrificed following optical/MRI imaging. The main organs (heart, liver, spleen, lung, and kidney) and the tumors were collected for the biodistribution of probes and histological analysis. The optical images for assessing the fluorescence distribution were obtained using the IVIS Spectrum system. Prussian blue staining was performed for the detection of iron in the tissue sections. Tissue morphology was verified by hematoxylin and eosin (HE) staining. The slices were examined using a digital microscope (Leica QWin, Wetzlar, Germany).

### Statistical analyses

All data were presented as the means ± standard deviation from at least a three sample replicates. Statistical comparisons were conducted using a Student's t-test or paired t-test and GraphPad Prism 5 software (LaJolla, CA, USA). A statistically significant difference was defined as a value of P < 0.05.

## SUPPLEMENTARY FIGURES


