# Supplementary material for: Theranostic imaging of liver cancer using targeted optical/MRI dual-modal probes
Source: Oncotarget. 2017 Feb 23;8(20):32741–51. doi: 10.18632/oncotarget.15642 (PMC5464824; doi:10.18632/oncotarget.15642)
Supplement: Supplementary file 1 [file oncotarget-08-32741-s001.pdf]

Theranostic imaging of liver cancer using targeted optical/MRI dual-modal probes

SUPPLEMENTARY FIGURES

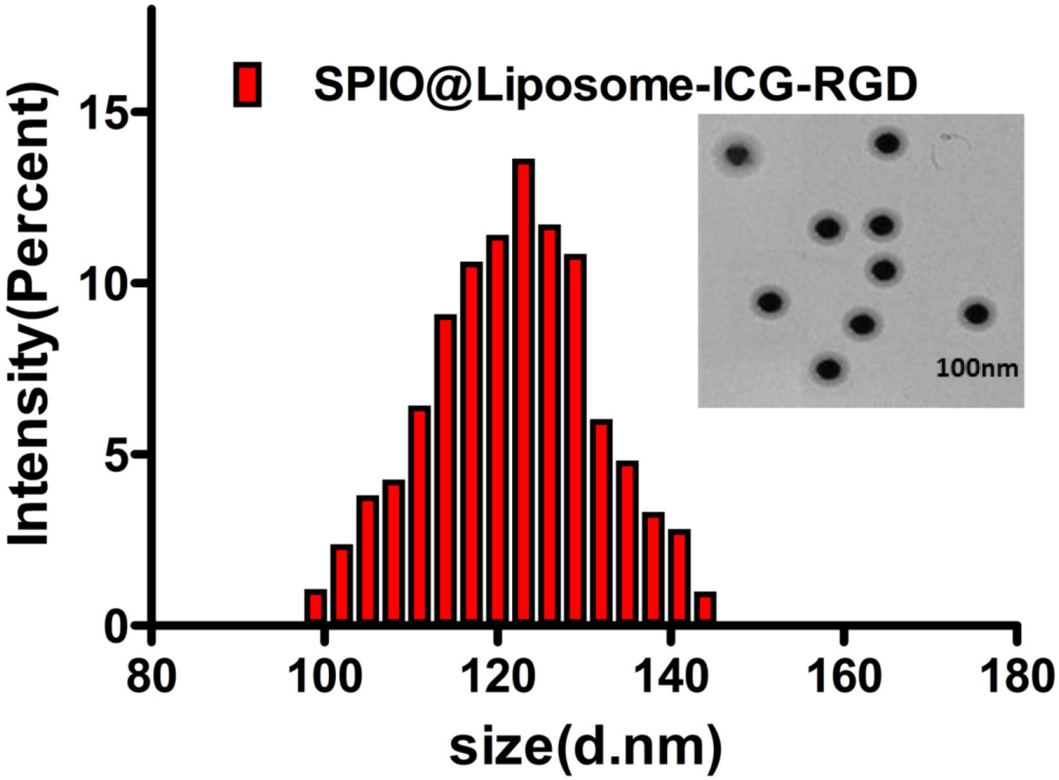

Supplementary Figure 1: Volume-based hydrodynamic size distribution of SPIO@Liposome-ICG-RGD NPs. Inset, TEM image of the probes (scale bar100 nm).

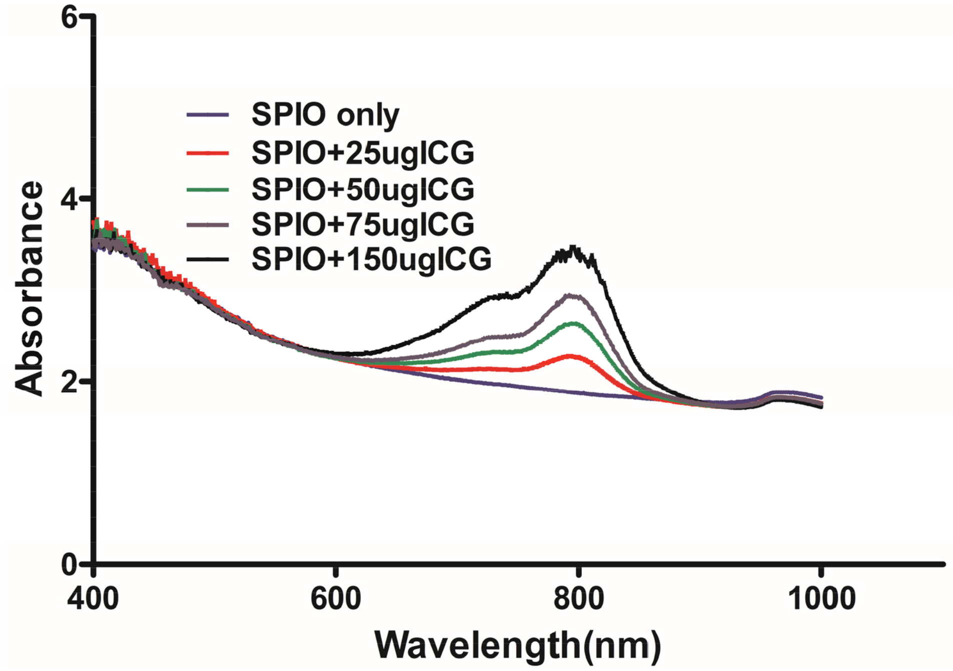

Supplementary Figure 2: UV-vis absorption spectra of SPIO@Liposome-ICG-RGD NPs with various amount of ICG. SPIO was kept constant (250 ug/mL).

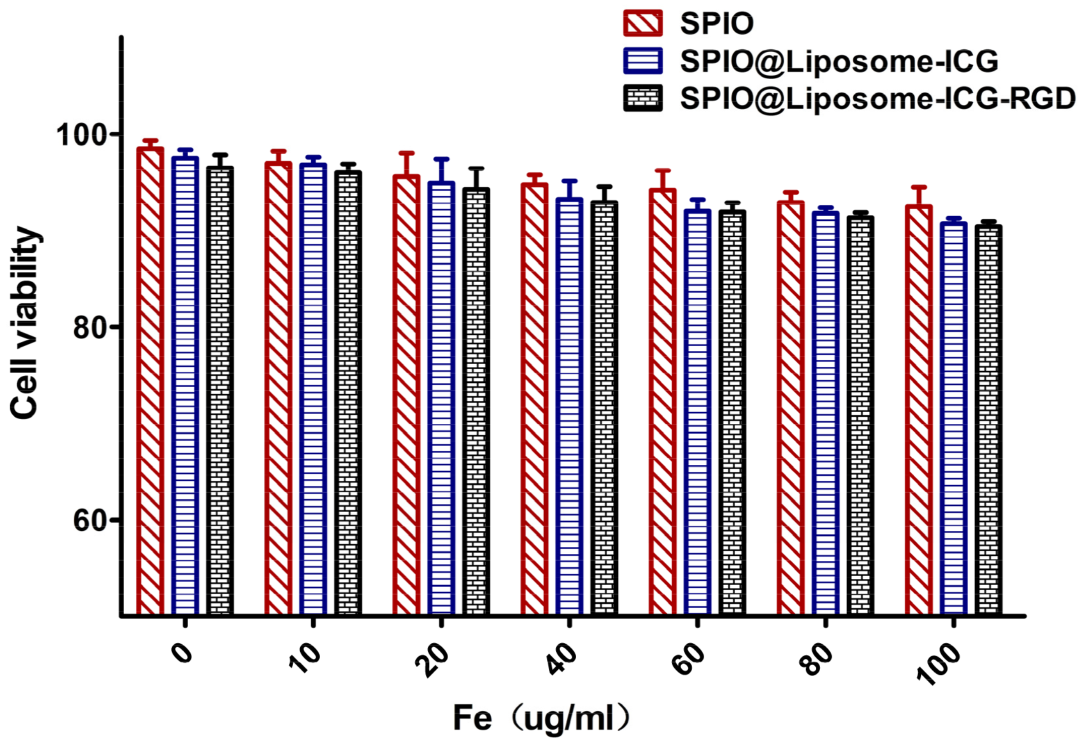

Supplementary Figure 3: The *in vitro* cytotoxicity and specificity evaluation of different nanoprobes. Cell-viability assays (The HepG2 cells) were applied to compare the SPIO, SPIO@Liposome-ICG and SPIO@Liposome-ICG-RGD over various concentrations. No statistically significant differences were found in the cell viability. Experiments were run in triplicate.

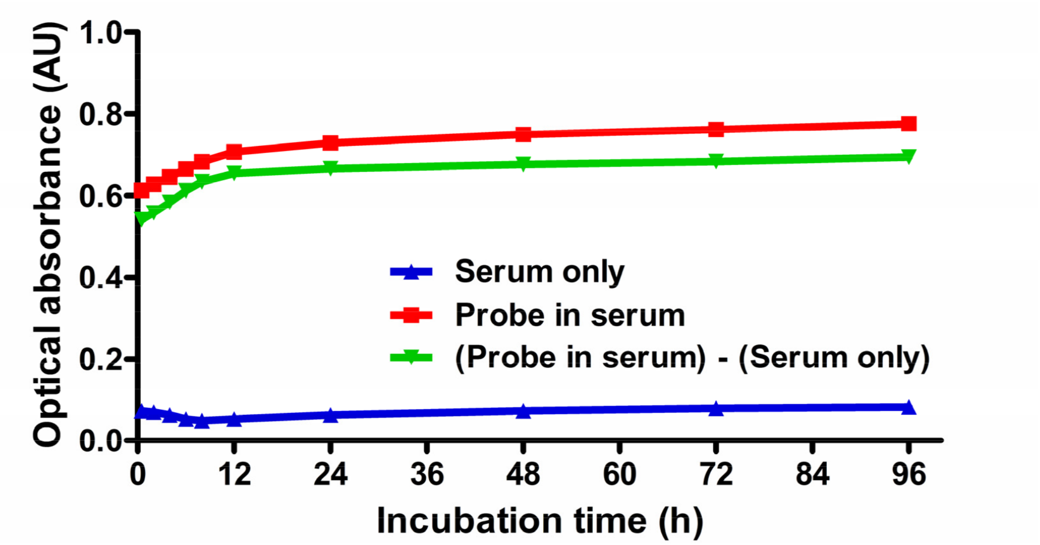

Supplementary Figure 4: The optical stability assessment of the probe in serum. The probe was added into mouse serum (total volume: 1 mL; the probe volume: 20 ul). We monitored the optical absorbance of the solution at 825 nm at various time points for 96 h. The control (serum only, blue curve) and the experiment (probe in serum, red curve) solution showed a slight increase of absorbance over time, which was likely due to the evaporation of the water from the sample. The subtraction (green curve) indicated a stable optical absorbance.

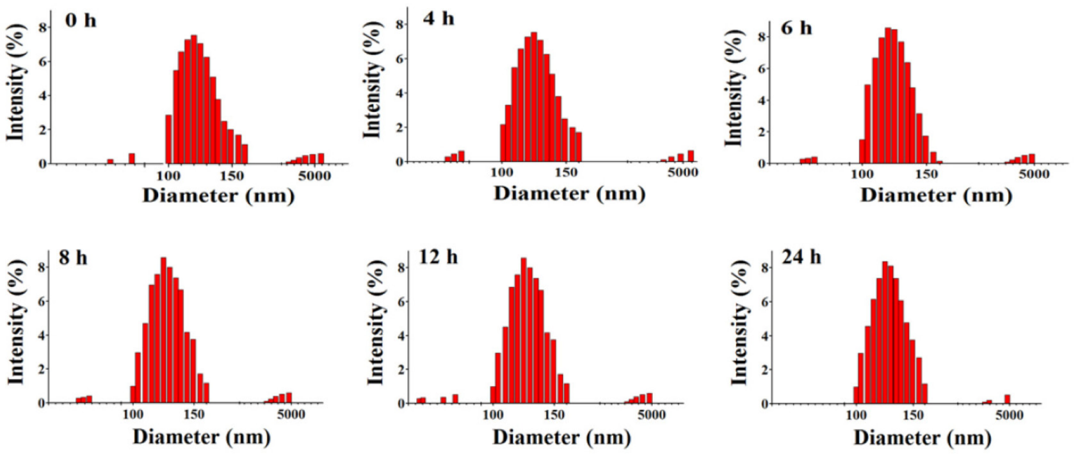

Supplementary Figure 5: Stability of the SPIO@Liposome-ICG-RGD hydrodynamic size during serum incubation. The probes were incubated with 50% PBS/50% mouse serum for 24 h at 37°C. Hydrodynamicmeasurements of the samples taken with a dynamic light scattering instrument indicated that thehydrodynamic size of the probes is stable over the course of 24 h of serum incubation.

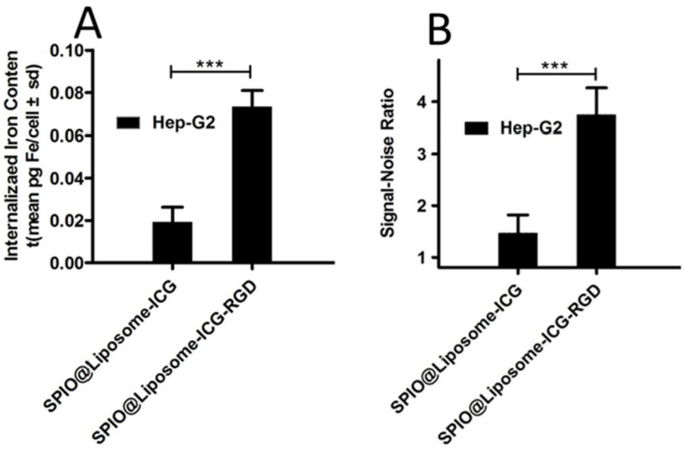

Supplementary Figure 6: *In vitro* evaluation of SPIO@Liposome-ICG-RGD specificity for liver tumor cells (Hep-G2) by measuring the iron content (A) and fluorescence intensity(showed as signal-noise ratio) (B). The modification of SPIO@ Liposome-ICG with RGD (SPIO@Liposome-ICG-RGD) substantially enhances the uptake of nanoprobes by Hep-G2 liver tumor cells, validating the specificity of SPIO@Liposome-ICG-RGD for tumor cells *in vitro*. $^*p < 0.01$.

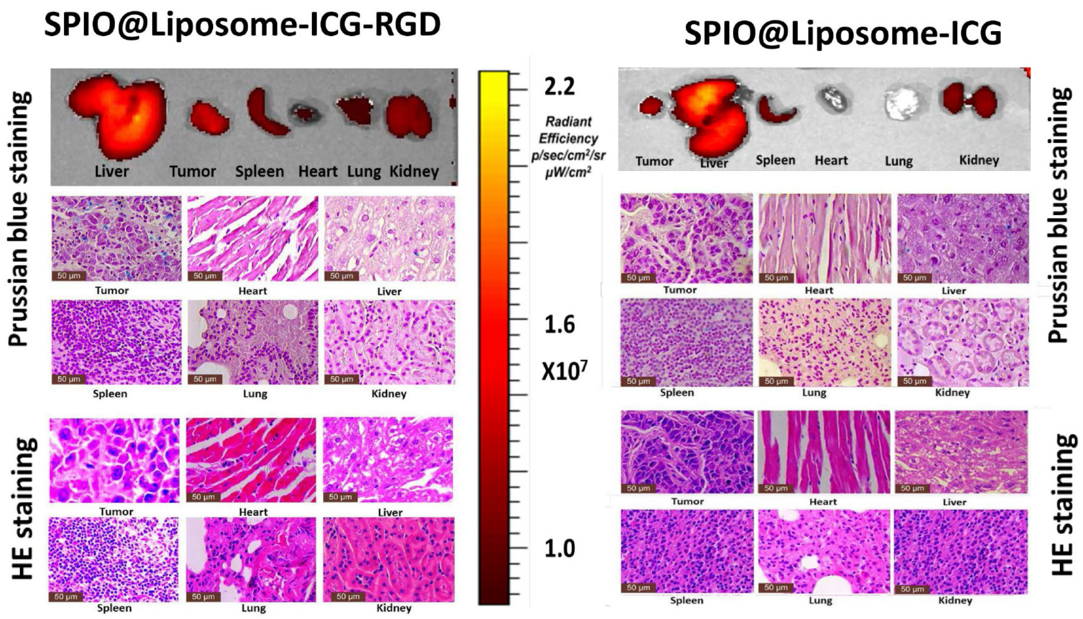

Supplementary Figure 7: The biodistribution of the probes and histology examination. The probes were selectively accumulated at the tumor sites other than any other organs. The probes (blue spot: tumor figure) in SPIO@Liposome-ICG-RGD group much more than it in the no-targeted groups.
